# Fascioliasis mimicking malignancy in an old patient

**DOI:** 10.1051/bmdcn/2019090207

**Published:** 2019-05-24

**Authors:** Mazaher Ramezani, Farhad Amirian, Masoud Sadeghi

**Affiliations:** 1 Molecular Pathology Research Center, Imam Reza Hospital, Kermanshah University of Medical Sciences Kermanshah Iran; 2 Medical Biology Research Center, Kermanshah University of Medical Sciences Kermanshah Iran; 3 Students Research Committee, Kermanshah University of Medical Sciences Kermanshah Iran

## Dear editor-in-chief

A 94-year-old lady along with her son, primary health care personnel, from a village in Songhor city in Kermanshah province, Iran referred to a physician in early 2016, with chief complaints of severe anorexia, weight loss, icterus, severe pruritus, and right upper quadrant pain. The symptoms and signs had been started a few months ago with insidious exacerbation. Drug history was unremarkable. Considering the patient’s old age, cancer workup was conducted. The complete blood count demonstrated prominent eosinophilia. Ultrasound examination reported bile duct dilatation and hepatomegaly. To rule out periampullary and pancreatic cancer, Computed Tomography (CT) scanning was requested, which demonstrated bile duct dilatation and a shadow in a choledochal canal with motility and echo in favor of parasite. The patient was referred to an infectious and tropical disease specialist. Fasciola hepatica egg was confirmed on the stool examination. In the past, she had a history of consumption of fresh plants growing near the river. The patient was prescribed Triclabendazole (6 tablets). She had been informed of obstructive symptoms of parasite if it could not be excreted. Fortunately, after drug ingestion, all the obstructive symptoms and signs were subsided. Pruritus continued for a few months. The patient had passed away in winter 2018, according to our last follow-up in July 2018.

Fascioliasis is an infestation of the liver by a fluke named Fasciola hepatica, which is a health problem in Iran. The number of human cases in Iran is underestimated. [1] Freshwater plant species, including watercress, especially in Iran, green leafy Nasturtium spp., and Mentha spp. may be the contributing factors for infestation. [1, 2] This zoonotic infestation is found in some provinces of Iran, including Kurdistan, Zanjan, Kermanshah, Mazandaran, Tehran, Azerbaijan, Guilan, Fars, and Khuzestan. [2, 3] Turkish researchers reported cases of Fascioliasis mimicking malignancy. [4, 5] One case was a 48-year-old lady with abdominal pain, fatigue, vomiting, nausea, weight loss, and a tiny neuroendocrine tumor, which finally showed Fasciola hepatica infestation with clinical, laboratory, and imaging data. [4] Another study included two ladies (26- and 52-year-old) with abdominal pain and unremarkable physical examination and lab data, except for peripheral eosinophilia and liver masses, which were finally diagnosed as Fasciola hepatica histopathologically. [5] The present case was a mimicker of malignancy by considering weight loss, anorexia, and icterus in an old lady. Physicians must be aware of Fascioliasis in managing the patients with hepatic lesions, weight loss, and gastrointestinal symptoms in contaminated areas and consider it in the differential diagnosis of malignancy.
